# Relationship between TIA minus C0-7 angle and C2-7 SVA: analysis of 113 symptomatic patients

**DOI:** 10.1186/s12891-022-05301-0

**Published:** 2022-04-08

**Authors:** Kai Yang, Xiang-Yu Li, Yu Wang, Chao Kong, Shi-Bao Lu

**Affiliations:** 1 Department of Orthopedics, Xuanwu Hospital, Capital Medical University, No.45 Changchun Street, Xicheng District, Beijing, China; 2 National Clinical Research Center for Geriatric Diseases, Beijing, China

**Keywords:** Cervical sagittal alignment, Thoracic inlet angle, C2-7 SVA, C0-7 angle

## Abstract

**Background:**

Measurement of T1 slope (T1S) can be difficult due to the anatomical positioning of the shoulders. And thoracic inlet angle (TIA) was a morphological parameter and not changed by the position. We proposed a new parameter, TIA minus C0-7 angle (TIA-C07), to evaluate C2-7 SVA in order to overcome the T1S imperfection.

**Methods:**

This was a retrospective radiological analysis of symptomatic subjects. The following cervical parameters were measured: Cervical lordosis angle (CL), C0-7 angle (C0-7), occiput-C2 lordosis angle (O-C2), C2-7 sagittal vertical axis (C2–7 SVA), TIA and TIA-C07. The Pearson correlation test was calculated, and the stepwise multiple regression analysis was conducted to determine the best predictor for C2-7 SVA. A paired sample t test was used to compare the predicted and measured C2-7 SVA.

**Results:**

The mean age of 113 patients was 60.02 ± 9.67. The average O-C2, CL, C0-7, TIA, TIA-C07 and C2-C7 SVA was 29.24 ± 8.48°, 13.67 ± 11.22°, 42.91 ± 11.44°, 76.07 ± 9.54°, 33.16 ± 13.18° and 21.34 ± 11.42 mm. The predictive formula was founded: C2-7 SVA = 2.80 + 0.56 * (TIA—C07) (*R* = 0.645, R2 = 0.416). There was no statistical difference between the predicted and the measured C2-7 SVA (t = 0.085, *P* = 0.933).

**Conclusions:**

TIA and C0-7 mismatch may significantly impact cervical alignment, and a greater T1A-C07 was related to a greater degree of C2-7 SVA. TIA-C07 may be a more important predictor for C2-7 SVA.

**Supplementary Information:**

The online version contains supplementary material available at 10.1186/s12891-022-05301-0.

## Background

Cervical spondylosis is a common cause of spinal cord dysfunction and imposes a serious social burden [1]. Cervical sagittal alignment had correlations with symptoms of patients and outcomes of treatment [2, 3]. Although various cervical sagittal parameters have been proposed, C2-7 SVA, as the key parameter to evaluate cervical sagittal alignment balance, has an important effect on symptoms [4].

As one measure of symptom and disability status for prognostic purposes, Neck Disability Index (NDI) was widely used to assess self-rated disability in patients with neck pain [5]. Studies confirmed that increased C2–7 SVA was associated with increased NDI [2, 4]. Thus, achieving a normal C2-7 SVA is one important goal for cervical deformity correction and cervical spondylosis treatment to obtain a good outcome. However, it is difficult to assess C2-7 SVA directly during surgery. Different cervical parameters have different impacts on C2-7 SVA. Evaluating other parameters during operation may help to reconstruct normal C2-7 SVA or normal lordosis indirectly. Lee et al. demonstrated that C2-7 SVA, T1S, and C2-7 lordosis were correlated with each other [6]. Staub et al. predicted normal cervical lordosis via formula CL = T1S − 16.5° ± 2° [7]. Li et al. reported that CL had a significant correlation with T1S, and optimal ratio of CL and T1S was related with good radiology outcome in patients with laminoplasty [8]. A literature review also reported that T1S can affect the lordosis of cervical spine [9]. However, measurement of T1 slope can be difficult due to the anatomical positioning of the shoulders, especially in obese patients with thick thoracic trunks, which can obscure visualization of the T1 superior endplate on radiographs. Studies have shown that the sternum and T1 vertebral related parameters could be estimated in 11% of the X-ray scans [10] and that the reproducibility of the T1 related parameters was extremely low [11]. Some authors studied that C7 slope was used as a substitute for T1S [12, 13]. But those authors also admitted that C7 slope was not a complete substitute.

In 2012, Lee et al. reported that thoracic inlet angle (TIA) was a morphological parameter and not changed by the position [6]. Subsequent studies showed that TIA is associated with some cervical parameters: such as T1S [14, 15]. Some studies showed that MRI was an optimal substitute for x-ray scans when measuring thoracic inlet alignment [16, 17].

Combined with the accuracy and invariance of TIA, we proposed a new parameter, TIA minus C0-7 angle, to evaluate C2-7 SVA in order to overcome the T1S imperfection. The purpose of our study was to assess the relationship between TIA minus C0-7 angle and C2-7 SVA.

## Materials and methods

### Patient population

We retrospectively evaluated consecutive patients who presented with cervical spondylosis between January 2019 and June 2021. Inclusion criteria was: Patients with neck pain, radiculopathy symptoms, and/or myelopathy, patients that received both a cervical MRI and a cervical radiograph during a single visit and aged 18 years or older. Exclusion criteria was: 1) previous surgery on the cervical spine. 2) cervical spine deformity resulting from fracture, tumor, infection, or congenital abnormality. 3) neuromuscular disease, or inflammatory arthritis including ankylosing spondylitis and rheumatoid arthritis. Evaluators were blinded to patient demographic and clinical characteristics. Another recruited 26 asymptomatic cases who had cervical MRI and cervical radiograph were used to verify regression equation. This study was approved by the ethical review board at our institution. Informed consent was obtained from all subjects.

### Cervical parameters measurement

The cervical sagittal alignment parameters were measured by PACS system. The following parameters were measured through lateral X-ray: (1) occiput-C2 lordosis angle (O–C2 angle, measured by the angle subtended between McGregor’s Line and along the inferior endplate of C2); (2) C2-7 angle defined as the CL angle (CL, angle subtended by lines drawn along the posterior vertebral bodies of C2 and C7); (3) C0-7 angle measured by the angle subtended between McGregor’s line (connecting the posterior part of hard palate and the most caudal part of occiput) and along the inferior endplate of C7; (4) C2-7 SVA defined as the distance between the C2 plumb line and the posterior C7 upper endplate.

**Fig. 1:**
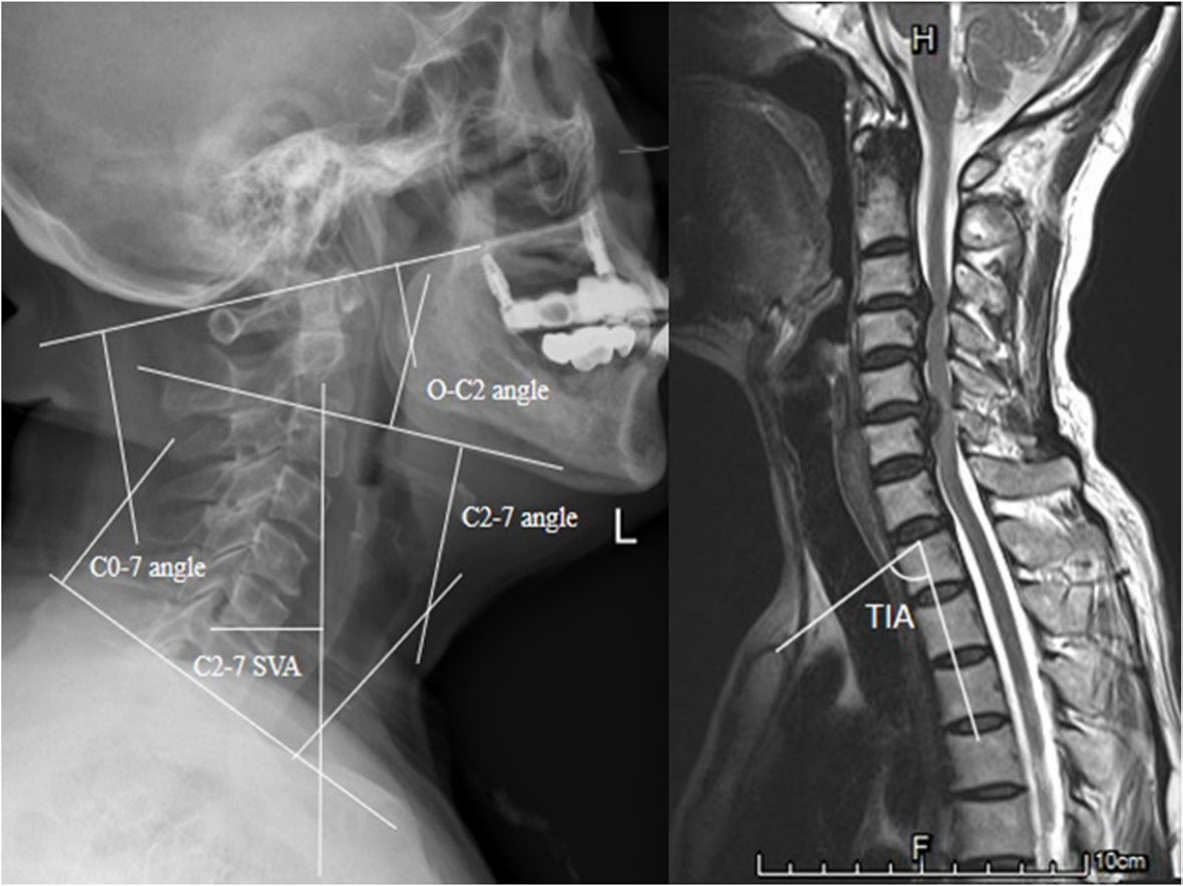
Measurements of different parameters

As a constant morphological parameter, not influenced by the posture, we defined TIA as an angle formed by a line from the center of the T1 upper end plate (T1UEP) vertical to the T1UEP and a line connecting the center of the T1UEP and the upper end of the sternum. TIA was measured through T2-weighted sagittal MRI. Measurements of parameters were shown in Fig. 1. TIA minus C0-7 angle was simplified as TIA-C07. All data were measured and calculated by two spine surgeons.

### Statistical analysis

The data was statistically treated with SPSS 24.0 software (IBM Corp, New York, USA). Measurement data was expressed in terms of mean ± SD. The correlations between the parameters were analyzed with the Pearson correlation coefficient. Stepwise multiple linear regression was used to analyze independent variable to affect C2-7 SVA and calculate regression equation. Paired t-test was used to compare measured values of C2-7 SVA with predicted values by regression equation in 26 cases. *P* < 0.05 was considered significant.

## Results

### Demographic data and sagittal parameters

In this retrospective study, we initially analyzed 118 consecutive patients, of whom 2 were excluded due to cervical spine infection, and 3 were excluded due to previous cervical spine surgery. 113 cases were included for the final analysis. The mean age was 60.02 ± 9.67 (range, 35 to 83 years old). The average values were as follows: O-C2, 29.24 ± 8.48°, CL, 13.67 ± 11.22°, C0-7, 42.91 ± 11.44°, TIA, 76.07 ± 9.54°, TIA-C07, 33.16 ± 13.18°, C2-7 SVA, 21.34 ± 11.42 mm (as shown in Table 1).

**Table1:** General information of patients (*N* = 113)

Parameters	Average value (Mean ± SD)
Sex, Female: male	54:59
Age (years)	60.02 ± 9.67
O-C2 (degrees)	29.24 ± 8.48
CL (degrees)	13.67 ± 11.22
C0-7 (degrees)	42.91 ± 11.44
TIA (degrees)	76.07 ± 9.54
TIA-C07 (degrees)	33.16 ± 13.18
C2-7 SVA (mm)	21.34 ± 11.42

### Correlation Among Demographic Data and Radiographic Parameters

To explore the correlation between each pair of demographic and radiographic parameters, we performed a correlation coefficient test. We found no correlation between age and other parameters. CL was significantly correlated with O-C2, C0-7, TIA, TIA-C07 and C2-7 SVA (*P* < 0.05). With regard to the focus of the research, C2-7 SVA was significantly correlated with O-C2 (*r* = -0.187, *P* = 0.047), CL (*r* = -0.259, *P* = 0.006), C0-7 (*r* = -0.393, *P* < 0.001), TIA (*r* = 0.421, *P* < 0.001) and TIA-C07 (*r* = 0.654, *P* < 0.001). which were included in next multiple regression analysis. There was no correlation between C2-7 SVA and age. All data was shown in Table 2.

**Table 2:** Correlation analysis between different parameters

Characteristics	CL	C2-7 SVA	O-C2	C0-7	TIA	TIA-C07
Age	0.123 0.194	0.080 0.399	-0.126 0.184	0.027 0.773	0.133 0.161	0.072 0.447
CL		**-0.259** **0.006**	**-0.352** **0.000**	**0.720** **0.000**	**0.295** **0.002**	**-0.411** **0.000**
C2-7 SVA			**-0.187** **0.047**	**-0.393** **0.000**	**0.421** **0.000**	**0.645** **0.000**
O-C2				**0.396** **0.000**	-0.094 0.542	**-0.411** **0.000**
C0-7					**0.220** **0.019**	**-0.08** **0.000**
TIA						**0.533** **0.000**

### Multiple regression analysis between C2-7SVA and potential factors

Multiple linear regression analysis was used to model the relationship between C2-7 SVA and potential factors by fitting a linear equation to the data. In our study, we regarded C2-7 SVA as a dependent variable and took O-C2, CL, C0-7, TIA and TIA-C07 as independent variables. The results were shown in Tables 3 and 4. A linear regression equation was established: C2-7 SVA = 2.80 + 0.56 * (TIA-C07) (*R* = 0.645, R^2^ = 0.416).

**Table 3:** Stepwise multiple regression analysis

Model	R	R^2^	Adjusted R^2^	Standard Error of estimate	R^2^change	F	Sig F change
1	0.645	0.416	0.411	8.765	0.416	79.231	0.000

a. Predictors (constant): TIA-C07

b. Dependent Variable: C2-7 SVA

**Table 4:** The coefficient and constant of predictive formula

Model		Regression coefficient	Standard Deviation	Standardized coefficient	t value	P value	Tolerance	VIF
1	Constant	2.803	2.240		1.251	0.214		
	TIA-C07	0.559	0.063	0.483	8.901	0.000	1.000	1.000

*VIF* variance inflation factor

### Verification of the accuracy of the regression equation

**Fig. 2:**
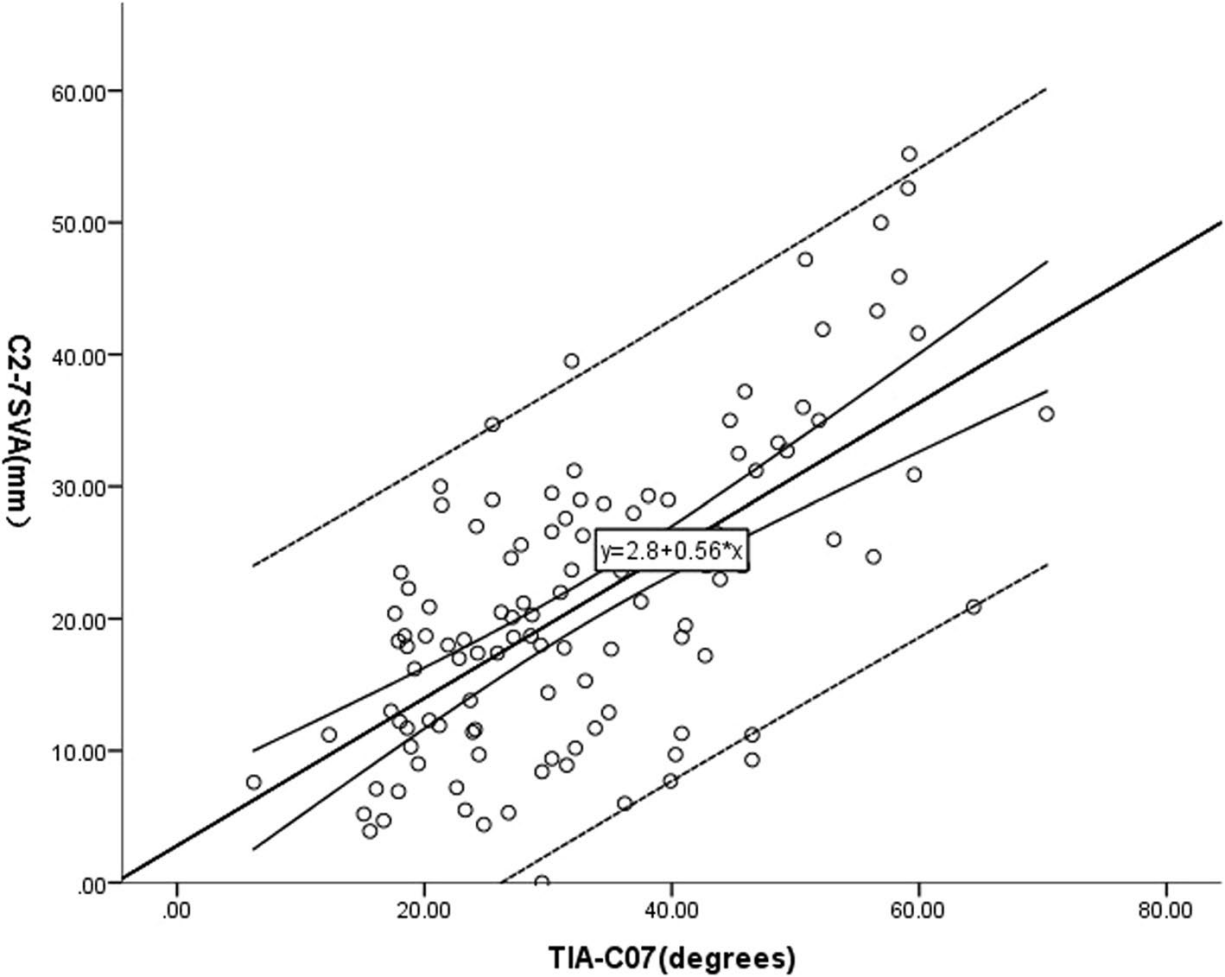
The scatter plot to show a linear relationship between C2-7 SVA and TIA-C07

**Fig. 3:**
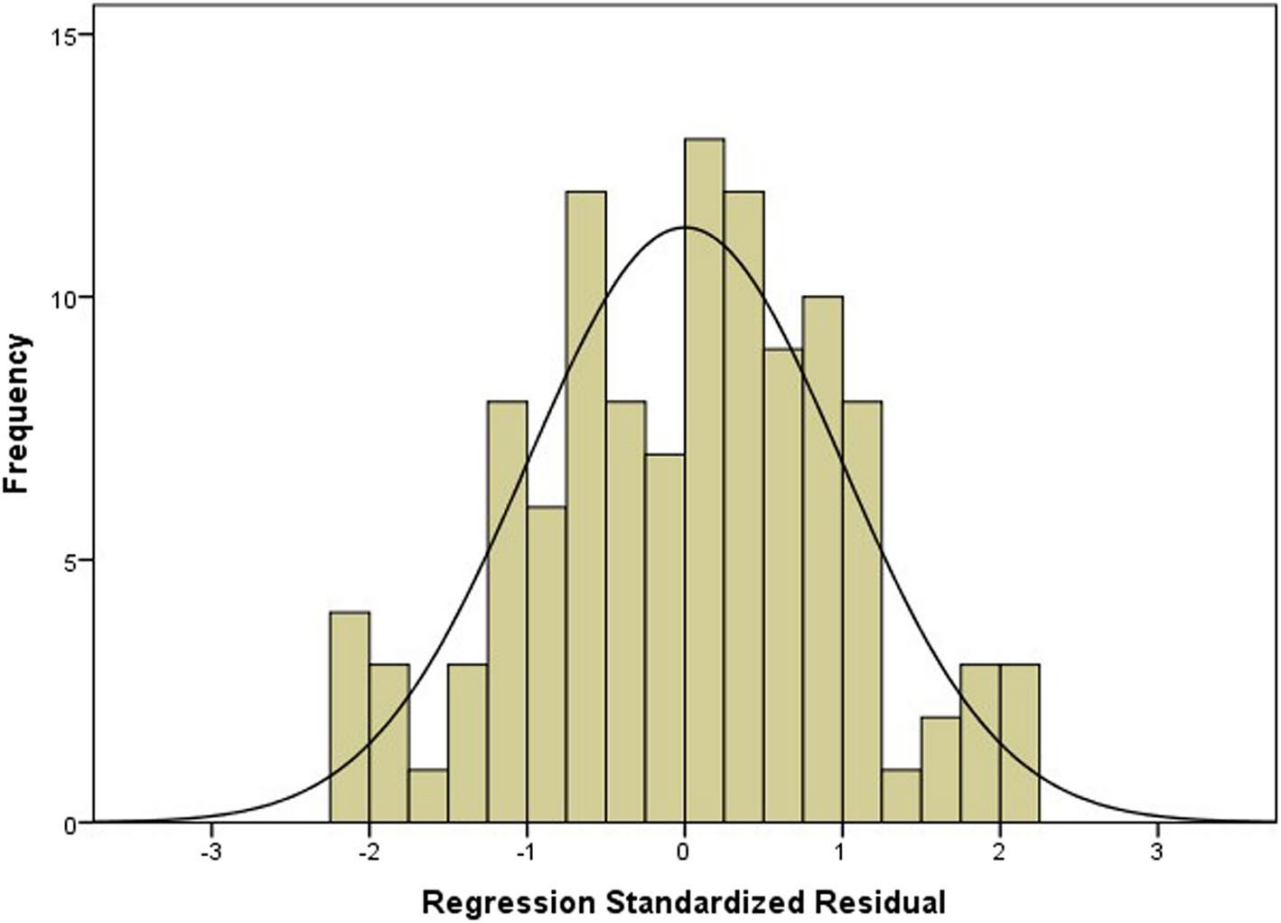
Histogram of residual analysis

According to the multiple linear regression analysis, only one model was formed. In the collinearity analysis, the tolerance was greater than 0.1 and the variance inflation factor (VIF) was less than 5, which supported the independence of factors contributing to the regression equation (as shown in Table 4). The scatter plot showed a linear relationship between C2-7 SVA and TIA-C07(as shown in Fig. 2). The histogram showed that the residuals of standard regression tended to be normally distributed (Fig. 3).

Another 26 cases were used to verify the regression equation practically. All data was shown in Table 5. The data was input into regression to get predicted C2-7 SVA (Table 5). The mean measured C2-7 SVA was 17.90 ± 12.37 mm. The mean predicted C2-7 SVA was 17.67 ± 6.57 mm. There was no significant difference (*P* = 0.933) between the predicted C2-7 SVA and the measured C2-7 SVA (Table 6).

**Table 5:** Data of cases for verification

Number	Gender	Age	C0-7	TIA	Measured C2-7 SVA	Predicted C2-7 SVA
1	F	53	38.9	61.7	6.4	15.57
2	F	65	45.1	69.3	4.7	16.35
3	F	63	72.2	93.9	30.2	14.95
4	F	49	51.6	61.8	2.9	8.51
5	F	38	30.0	54.8	19.9	16.69
6	F	62	34.9	57.1	7.6	15.23
7	F	66	62.9	80.1	23.0	12.43
8	F	60	52.5	58.7	16.0	6.27
9	F	70	30.8	65.7	4.3	22.34
10	F	58	48.1	62.6	18.7	10.92
11	F	62	41.1	86.1	24.0	28.00
12	F	54	68.7	72.0	25.9	4.65
13	F	60	22.0	69.4	27.5	29.34
14	F	70	37.1	67.1	6.0	19.60
15	M	65	32.2	59.2	13.7	17.92
16	M	31	49.1	73.1	10.9	16.24
17	M	70	55.9	88.3	34.7	20.94
18	M	53	33.6	80.2	25.7	28.90
19	M	52	43.6	71.8	3.4	18.59
20	M	65	35.0	53.3	14.7	13.05
21	M	68	64.9	88.3	-2.9	15.90
22	M	71	56.6	84.4	26.5	18.37
23	M	65	46.6	83.4	13.1	23.41
24	M	68	29.8	63.8	50.3	21.84
25	M	63	47.9	70.8	25.6	15.62
26	M	65	24.6	70.4	32.6	28.45

*F* female, *M* male

**Table 6:** Comparison between measured C2-7 SVA and predicted C2-7 SVA

Parameters	Average value (Mean ± SD)
Age(years)	60.23 ± 9.76
Female: male	14:12
Measured C2-7 SVA (mm)	17.90 ± 12.37
Predicted C2-7 SVA (mm)	17.67 ± 6.57
*t* value	0.085
*P* value	0.933

## Discussion

A number of studies demonstrated that the C2-C7 SVA is an important parameter in determining cervical sagittal balance [2, 15–17]. Iyer et al. showed that a high preoperative C2-7 SVA was an independent predictor of a high neck disability index score [18]. In addition, Tang et al. noted that a larger C2-7 SVA was directly and negatively correlated with the 36-item short-form health survey [2]. Oe et al. described how C2-7 SVA negatively influenced the results of the EuroQol-5D short-form health survey, including mobility, self-care, usual activities, pain or discomfort, and anxiety or depression [19]. C2-7 SVA was correlated with different parameters. Previous studies assessing factors influencing C2-7 SVA mainly concentrated on T1S, C2-7, C0-7 and O-C2 [8, 10, 14, 15, 20]. Hyun SJ et al. got an equation: C2-7 SVA (mm) = 1.4178 * (T1S-CL) + 8.852 [21]. Shao ZX et al. considered that C2-7 SVA was affected by different parameters and attained an equation: C2-7 SVA (mm) = 0.38 * BMI—0.73 * O-C2 + 0.15 * CL + 0.18 * T1S—6.53 [22]. These studies showed that T1S was a very important parameter to affect C2-7 SVA.

Unfortunately, T1UEP is not always easily visualized on radiographs due to anatomical interference from the shoulders and thoracic trunk. Tamai K et al. report that 62.2% of T1UEP were invisible on cervical radiographs in sitting position [13]. Park et al. reported an 11% visualization rate of the T1UEP in their series of cervical spine radiographs in 200 patients [10]. Ye reported T1UEP was only visualized in 31% of weightbearing sitting radiographs [12]. Furthermore, T1S was significantly influenced by flexion and extension of the neck [23, 24].

In order to overcome the drawbacks of T1S, surgeons choose to use TIA because it is not changed by the position and easily measurable on CT or MRI [6, 14]. Cheng J reported that MRI could be useful to evaluate TIA in patients with cervical spondylosis [17]. So, TIA value was accurate and reproducible relatively. Lee et al. studied that the mean TIA was 69.5 ± 8.6° and TIA was correlated with T1S, C2-7 and O-C2 [14]. Zhu et al. reported that the mean TIA was 67.87 ± 7.87° and TIA was correlated with CL [24]. In turn, the authors got a predictive formula of CL: CL = 0.417 * TIA − 11.193 [24]. Lee SH et al. reported that TIA was more significantly related with T1S than TK and was correlated with C0-7 and C2-7 [14]. Thus, TIA may influence C2-7 SVA by affecting T1S and CL. In the present study, TIA was correlated with C2-7 SVA, CL and C0-7. This result verified the effect of TIA on C2-7 SVA. As TIA increased, C2-7 SVA may increase correspondingly.

Some studies proved that CL was an important parameter to affect C2-7 SVA [6, 21, 24]. Some authors studied that CL had no correlation with C2-7 SVA [17, 18]. In the current study, CL was negatively related with C2-7 SVA (*R* = -0.259, *P* = 0.006). Some studies have clarified the correlation between O-C2 and C2-7 SVA [18, 21, 22]. So did our results. However, Ikeda et al. reported that O-C2 had no direct relationship with C2-7 SVA but the change of O-C2 can compensate the decrease of CL to affect C2-7 SVA and keep horizontal gaze [25]. In the present study, we found significant correlations between O-C2, CL and C0-7. Moreover, C0-7 angle showed stronger correlation with CL. Hardacker and Lee also described stronger correlation of lower cervical spine angle than upper cervical spine angle with C0-7, despite less lordosis in the lower cervical spine [14, 26]. The authors thought that this phenomenon could be a result of possible reciprocal influence of upper and lower cervical alignments. Patwardhan et al. noted that increased C2-7 SVA caused flexion of the lower cervical (C2-7) segments and hyperextension of the suboccipital (C0-C2) segments, Thus, CL and O-C2 may have a compound effect on C2-7 SVA [27]. When C2-7 SVA was studied, Both O-C2 and CL should not be ignored. In our study, there was negatively stronger correlation between C2-7 SVA and C0-7 than between O-C2, CL and C2-7 SVA.

**Fig. 4:**
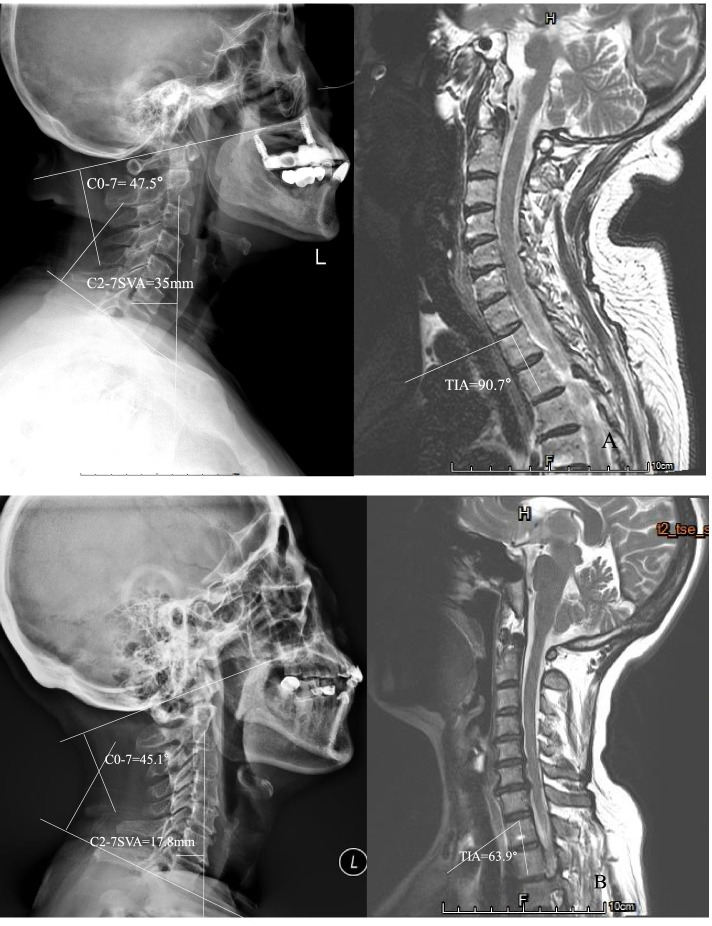
Although two patients had similar C0-7(A:47.5°, B: 45.1°), C2-7 SVA (35 mm) of patient A was larger than patient B (17.8 mm) because of patient A with larger TIA-C07 (43.2°) than patient B (18.8°)

Based on the above analysis, we introduced TIA-C07 as an independent variable. In Pearson correlation analysis, we found correlations between C2-7 SVA and O-C2, CL, C0-7, TIA and TIA-C07. Moreover, TIA-C07 had a stronger relation with C2-7 SVA than others. By multiple linear regression analysis, only TIA-C07 was reserved. We got a regression equation: C2-7 SVA = 2.80 + 0.56 * (TIA-C07). By the verification of statistics and 26 cases, we affirmed the rationalization of the equation. It meant that TIA and C07 mismatch may significantly impact cervical alignment (Fig. 4). The present result indicated that a greater mismatch between TIA and C0-7 was associated with a greater degree of cervical malalignment. Similar to PI-LL, as TIA increases, C0-7 also needs to be increased accordingly to maintain a normal cervical alignment. TIA was a morphological parameter, C0-7 was a change parameter and a compensatory factor. Our finding can provide a guidance for cervical spine surgery. Before surgery of the cervical spine, we could plan the corrected degree of cervical lordosis according to predictive formula in order to get ideal C2-7 SVA. This may be helpful during surgical planning.

In this study, the sample size was small. There was no case of severe cervical deformity such as ankylosing spondylitis. The average age was relatively older. The analyzed 113 subjects came from symptomatic patients. The next study in younger and asymptomatic people may be needed. Even so, we firstly founded the relationship between TIA-C07 and C2-7 SVA.

## Conclusions

The correlation between C2-7 SVA and TIA-C07 was firstly founded. TIA and C0-7 mismatch may significantly impact cervical alignment, and a greater TIA-C07 was related to a greater degree of C2-7 SVA. An individual with large TIA required large C0-7 to preserve physiologic sagittal balance of the cervical spine. TIA-C07 may be a more important predictor for C2-7 SVA. TIA-C07 can be used as a reference for estimating the normal value of C2-7 SVA. The results of this study may provide a useful reference for further studies.

## Supplementary Information

**Additional file 1.**

## Data Availability

All data generated or analyzed during this study are included in this published article and its supplementary information files.

## Abbreviations

TIA: Thoracic Inlet Angle
TIA-C07: TIA Minus C0-7 Angle
C2-7 SVA: C2-7 Sagittal Vertical Axis
CL: Cervical Lordosis
C0-7: C0-7 angle
O-C2: Occiput-C2 lordosis angle
TIA-C07: Thoracic inlet angle and TIA minus C0-7
T1S: T1 Slope
T1UEP: T1 Upper End Plate
VIF: Variance Inflation Factor
